# Effects of chronic noise on glucose metabolism and gut microbiota–host inflammatory homeostasis in rats

**DOI:** 10.1038/srep36693

**Published:** 2016-11-04

**Authors:** Bo Cui, Zhihui Gai, Xiaojun She, Rui Wang, Zhuge Xi

**Affiliations:** 1 Department of Occupational Hygiene, Tianjin Institute of Health and Environmental Medicine, Tianjin, China; 2 School of Medicine and Life Sciences, University of Jinan-Shandong Academy of Medical Sciences, Jinan, China; 3 Shandong academy of occupational health and occupational medicine, Shandong academy of medical sciences, Jinan, China

## Abstract

Chronic noise exposure has been implicated in increased risk of diabetes. However, there is limited experimental evidence of the mechanisms linking chronic noise stress and glucose metabolism. We addressed this in the present study by examining glucose metabolism, immune response, and changes in gut microbiota/host inflammatory homeostasis in rats exposed to noise for 30 consecutive days. Chronic noise exposure increased blood glucose and corticosterone levels for at least 14 days after cessation of noise. Stressed rats also exhibited elevated levels of glycogen and triglyceride in the liver and impaired hepatic insulin production via insulin-induced insulin receptor/insulin receptor substrate 1/glycogen synthase kinase 3β signalling, which persisted for 3–14 days after cessation of noise exposure. Chronic noise altered the percentage of Proteobacteria and Actinobacteria in the gut, increasing *Roseburia* but decreasing *Faecalibacterium* levels in the cecum relative to controls. Immunoglobulin A, interleukin 1β, and tumor necrosis factor α levels were also elevated in the intestine of these animals, corresponding to noise-induced abnormalities in glucose regulation and insulin sensitivity. These results suggest that lifelong environmental noise exposure could have cumulative effects on diabetes onset and development resulting from alterations in gut microbiota composition and intestinal inflammation.

The human population is increasingly being exposed to environmental noise from many sources including traffic, media, and household appliances. Long-term noise exposure is considered as a health hazard^1^ and has been linked to non-auditory effects, including Alzheimer’s disease-like neuropathology^2^,^3^ and metabolic disorders^4^. Recent epidemiological studies have shown that chronic noise exposure is associated with increased risk of adiposity^5^,^6^, higher levels of cholesterol^7^ and development of diabetes^8^. A more recent experimental study also indicated that noise exposure at 95 dB SPL caused insulin resistance in mice, which was prolonged by longer noise exposure^9^. However, detailed experimental evidence for the mechanisms underlying these associations is limited.

Noise acts as a stressor that hyperactivates the sympathetic autonomic nervous system and activates the hypothalamic–pituitary–adrenal axis, which increases cortisol levels^1^,^10^,^11^,^12^ and potentially alters metabolism^13^. Elevated glucocorticoid levels inhibit insulin secretion by pancreatic β cells and reduce insulin sensitivity in the liver, skeletal muscle, and adipose tissue^14^, thereby increasing the risk of diabetes^15^,^16^, a metabolic disease characterised by insulin resistance and low-grade inflammation that is one of the leading causes of disability and mortality in modern societies. Apart from individual risks, its incidence may be influenced by environmental factors such as air and noise pollution. Recently, interest has surged regarding a possible role for intestinal microbiota as contributors to the increased prevalence of metabolic syndromes, including type 2 diabetes^17^. Gut microbiota may play an important role in weight regulating and may be partly responsible for the development of metabolic disorders^18^,^19^. The human body harbours a vast array of microbes collectively referred to as microbiota, and there is increasing evidence that their relationship to systemic inflammation underlies diabetes pathogenesis^17^. Psychological stressors have been shown to alter the composition of gut microbiota, which in turn negatively affects glucose metabolism, inflammation, and insulin activity, potentially leading to obesity and diabetes^20^. Noise, like other psychological stimuli, causes metabolic alterations and inflammation, and may perturb glucose metabolism.

We speculated that commensal microbiota play a role in noise stress-induced metabolic disorders. To test this hypothesis, we investigated the effects of chronic noise exposure on glucose regulation, microbiota composition, and inflammation in rats. Given the persistent effects of chronic noise on glucose homeostasis^9^, it is important to explore the potential aftereffects of noise exposure on the development of metabolic disorders.

## Results

### Chronic noise exposure increases blood glucose and corticosterone (CORT) levels

**Figure 1:**
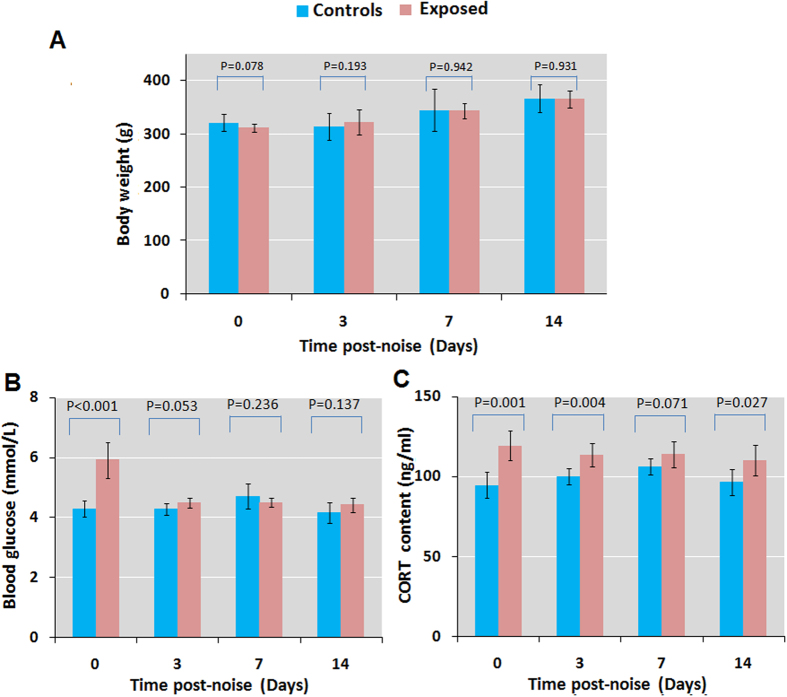
Effects of chronic noise exposure on body weight and blood glucose and CORT levels. (**A**–**C**) Comparison of body weight (**A**), blood glucose level (**B**), and blood CORT level (**C**) after noise exposure in control and noise-exposed rats at indicated time points. Bars represent mean ± SD (n = 7 or 8 per condition).

We examined changes in body weight and measured blood glucose and CORT levels by enzyme-linked immunosorbent assay (ELISA) in rats exposed to noise for various periods. Noise exposure had no effect on body weight (Fig. 1A); however, blood glucose level was increased relative to controls on the day after exposure before declining to the baseline 3 days after the end of the 28-day noise exposure period (Fig. 1B). ELISA results indicated that CORT level was elevated by 26.2%, 13.3%, and14.0% on days 0, 3, and 14, respectively (Fig. 1C). These results indicate that chronic noise exposure induces a persistent stress response and glucose dysregulation.

### Chronic noise exposure decreases hepatic insulin sensitivity

**Figure 2:**
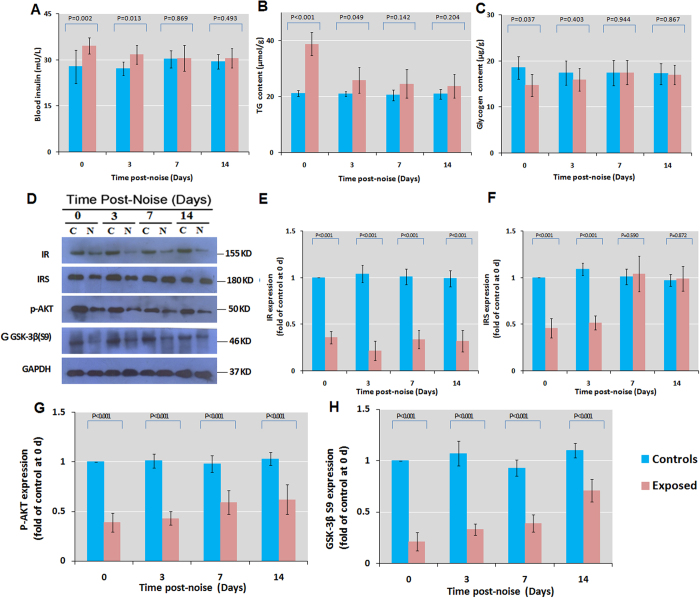
Chronic noise exposure dysregulates hepatic insulin sensitivity in rats. (**A**–**C**) Quantification of blood insulin and glycogen levels and liver TG level by ELISA at indicated time points following noise exposure. (**D**) Western blot analysis of IR, IRS-1, p-AKT, and GSK-3β (S9) levels in the liver under control (**C**) and chronic noise exposure (N) conditions. Glyceraldehyde 3-phosphate dehydrogenase (GAPDH) was used as a loading control. (**E**–**H**) Quantification of protein band densities shown in panel (**D**). Data show percent change relative to control samples. Bars represent mean ± SD (n = 6 per condition).

The effect of chronic noise exposure on insulin sensitivity was assessed by measuring blood plasma insulin, glycogen, and triglyceride (TG) levels by ELISA as well as liver insulin receptor (IR), IR substrate (IRS), and AKT and glycogen synthase kinase (GSK)-3β levels by western blotting. Exposure to noise increased insulin and TG concentrations, with corresponding reductions in liver glycogen that persisted for 0–3 days after noise cessation (Fig. 2A–C). IR and IRS levels were decreased after chronic exposure to noise; this trend persisted up to 14 days after the cessation of noise stimulation (Fig. 2D–F). Phosphorylated (p-)AKT and p-GSK-3β levels were downregulated in the noise-exposed group 0, 3, 7, and 14 days after exposure (Fig. 2D,G,H). These data indicate that chronic noise exposure causes persistent activation of IRS–AKT–GSK3β signalling in the liver, potentially contributing to insulin resistance.

### Chronic noise exposure alters the composition of gut microbial communities

**Figure 3:**
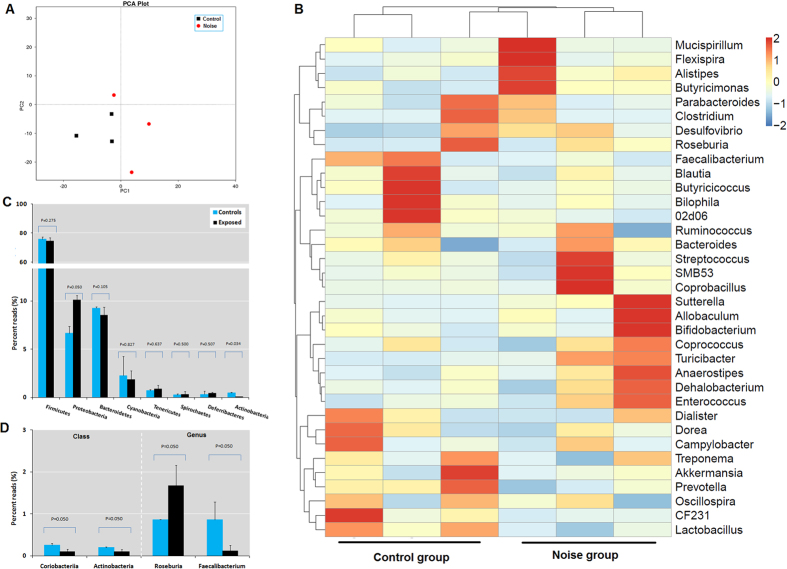
Effects of chronic noise exposure on gut microbiota composition in rats. Animals were exposed for 30 days to noise or not exposed and examined on day 30. (**A**) Weighted UniFrac principal coordinate analysis of the total population of the caecal microbiome in control and noise-exposed rats. (**B**) Cluster analysis of relative abundance of top 35 genera detected in caecal content of rats. (**C**,**D**) 16S rRNA gene sequencing of caecal content of rats at the phylum (**C**) and class and genus (**D**) levels. Data represent mean ± SD (n = 3 per condition).

A total of six faecal samples (three each from control and noise-exposed rats on day 0 after exposure) were collected for 16S rRNA gene sequencing. Each sample has 1276 OTUs and an average of 41 847 sequences. Stress can alter the composition of gut microbiota^17^; we therefore evaluated microbial community composition in the faecal samples. Weighted UniFrac principal coordinate analysis and cluster analysis of 16S rRNA sequencing results indicated that chronic noise exposure induced significant changes in the gut microbiota population at the phylum (Fig. 3A) and genus (Fig. 3B) levels. That is, Proteobateria and Actinobacteria numbers were altered after noise exposure (Fig. 3C). Furthermore, Coriobacteriia and Actinobacteria were depleted at the class level whereas Roseburia was enhanced and Faecalibacterium depleted at the genus level in the cecum of noise-exposed rats (Fig. 3D).

### Chronic noise exposure increases inflammatory responses

**Figure 4:**
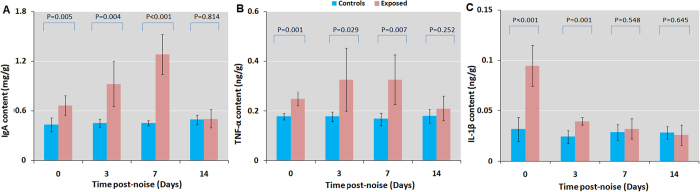
Chronic noise exposure induces inflammation in the intestine of rats at different time points following noise exposure. (**A**–**C**) IgA, Il-1β, and TNF-α levels following noise exposure, as determined by ELISA. Bars represent mean ± SD (n = 5 or 6 per condition).

To clarify the inflammatory events leading to metabolic dysfunction induced by chronic noise exposure, we measured immunoglobulin (Ig)A, interleukin (IL)-1β, and tumour necrosis factor (TNF)-α levels in the ileum by ELISA. Noise exposure induced increases in IgA, IL-1β, and TNF-α levels that persisted for at least 3 to 7 days after noise cessation (Fig. 4). These data indicate that chronic noise exposure causes persistent inflammation in the ileum, implying a mechanistic link to metabolic disorders such as diabetes and obesity.

## Discussion

Previous studies have reported that chronic noise exposure increases the risk of developing metabolic disorders such as diabetes and obesity^5^,^6^,^8^,^21^. The results presented here provide novel insight into the mechanism underlying this link, which involves noise-induced changes in glucose regulation, inflammation, and gut microbial community composition.

Exposure to road traffic noise is associated with a higher risk of incident diabetes^8^ and may also be involved in its development due to the effects of excess cortisol on glucose tolerance and insulin sensitivity^14^,^15^,^16^. Stress-induced changes in cortisol level are linked to altered fasting insulin and blood glucose concentrations^22^,^23^. A recent study indicated that noise-induced disturbances in the level of cortisol, a stress hormone, can contribute to the development of insulin resistance^9^. Our results showed that blood glucose and CORT levels were elevated in rats after chronic noise exposure. Furthermore, we found that the increase in blood CORT persisted for at least 7–14 days after the period of noise exposure ended, suggesting a cumulative impact of blood CORT on the risk of noise-induced insulin resistance.

Nighttime exposure to noise is associated with sleep disturbance and consequently, increased adiposity and risk of diabetes^24^,^25^,^26^,^27^,^28^,^29^. However, we did not observe significant differences in body weight between the two groups of rats after 30 days of noise exposure, and the body weights of noise-exposed animals were lower than those of controls on the last day of exposure, although all groups initially had similar body weights. This result is in accordance with a recent study reporting that animals exposed to chronic noise did not show a higher body weight than controls^9^. Our findings also downplay the possibility that increased body weight mediates the effects of daytime noise exposure on glucose metabolism, and suggest that daytime noise differs from residential road traffic noise and is unrelated to sleep disturbance, which can lead to a larger waist circumference and higher body mass index and percentage body fat^30^,^31^.

Psychological stress can interfere with carbohydrate metabolism and lead to insulin resistance^32^. Liver and skeletal muscle are the major targets of insulin-dependent glucose disposal^33^,^34^, in which insulin-induced signaling regulates major metabolic responses such as increased glucose transport into muscle and glycogen and lipid synthesis as well as decreased gluconeogenesis and glucose release from the liver. The activated insulin receptor (IR) engages and phosphorylates downstream signal proteins including those of the IR substrate (IRS) family that activate the phosphatidylinositol 3-kinase pathway and consequently, protein kinases such as Akt and GSK-3β. Previous studies have reported significant decreases in Akt phosphorylation and expression of glucose transporter 4 on the surface of skeletal muscle cells after exposure to various types of noise, which suggests that chronic noise exposure can lead to the development of insulin resistance^9^. Our finding of the lasting increase in liver TG and glycogen levels corresponding to an increase in blood insulin and CORT provides further evidence of noise-induced abnormalities in glucose regulation and insulin sensitivity. We also found that noise-exposed rats exhibited multiple effects related to insulin, including insulin-induced glycogen synthesis and persistent downregulation of IR and IRS1 as well as p-Akt and GSK-3β.

However, a previous study strongly suggested that the persistent dysregulation of insulin sensitivity cannot be explained solely by abnormalities in blood glucocorticoid level and that other mechanisms are involved in noise-induced insulin resistance^9^. Gut bacteria may play an important role in disorders such as obesity and diabetes. Chinese type II diabetes mellitus patients were found to have a moderate degree of gut microbial dysbiosis, decreased abundance of some universal butyrate-producing bacteria, and increased numbers of various opportunistic pathogens^35^. A recent study also showed that the proportion and diversity of intestinal microbiota are altered early in the prediabetes period^36^. A significant finding from our study was the alteration of gut microbiota composition as a result of chronic noise exposure. At the phylum level, the microbial community shifted from Proteobacteria to Actinobacteria. The former include gram-negative bacteria in the *Escherichia*, *Salmonella*, *Helicobacter*, and *Yersinia* genera. Noise exposure enriched *Roseburia* spp., which may be associated with weight loss and reduced glucose intolerance^37^, and reduced the levels of *Faecalibacterium* spp., which are associated with Crohn’s disease, obesity, and asthma^38^. Therefore, noise-induced disturbance of gut microbiota may mediate the progression from glucose dysregulation to metabolic syndromes.

Inflammation is related to stress-induced metabolic dysfunction, and inflammatory mediators such as cytokines and chemokines may contribute to insulin resistance at the tissue level^39^,^40^. In our recent study, we demonstrated reciprocal activation of pro-inflammatory cytokines and astrocytes after noise exposure, which could cause a positive feedback loop that results in central nervous system abnormalities^3^. Resident microbiota regulate gut inflammation^41^. Here we found that chronic noise exposure induced an intestinal inflammatory response in rats, as evidenced by persistent elevation of IgA, IL-1β, and TNF-α levels. Thus, chronic noise exposure directly or indirectly regulates gut microbiota–host inflammation homeostasis. IgA is produced in mucosal tissues and inhibits innate immune responses while modulating the composition and function of gut microbiota^42^. The increase in IgA level observed here indicates a shift in gut immune surveillance and an increase in inflammatory signalling. IL-1β and TNF-α levels were also increased in the intestine after noise exposure. TNF-α administration was previously found to increase TG and very low-density lipoprotein levels in rats and humans^43^ and inhibit insulin-stimulated glucose transport^44^. Insulin resistance is closely related to inflammation; in prediabetes and type 2 diabetes, cytokines are released that block insulin action, thereby lowering sensitivity and increasing resistance to insulin. Chronic inflammation is increasingly recognised as a factor in the aetiology of metabolic diseases^45^,^46^. Our findings provide evidence that chronic exposure to noise increases diabetes risk, possibly via dysregulation of the gut microbial community and resultant inflammation in the intestine.

The above-mentioned previous study described the relationship between environmental noise exposure and risk of diabetes development in a laboratory setting^9^. Our work confirms that dysregulation of the gut microbial community and resultant inflammation in the intestine caused by chronic noise exposure contributes to diabetes risk. However, one important limitation of this study was the small sample size for analysis of the 16S rRNA gene in microbiota, which may have led to statistical bias. In addition, the results of the microbiome were obtained at a single time point (at the end of the noise exposure), which does not provide information on long-term, noise-induced aftereffects in gut microbiota.

## Conclusions

Our results indicate that chronic noise exposure induces persistent abnormalities in glucose regulation accompanied by alterations in gut microbiota and host immune responses. Although the study examined a relatively small number of subjects, these findings nonetheless enhance our understanding of the etiological association between chronic noise exposure and metabolic dysregulation and suggest that exposure to environmental noise over a lifetime may have a cumulative effects on the development of metabolic syndromes.

## Materials and Methods

### Animals and experimental groups

In total, 64 male Wistar rats (Laboratory Animal Center, Institute of Health and Environmental Medicine, Tianjin, China) weighing 200–220 g used in this study and maintained in a room on a 12:12-h light/dark cycle (lights on from 06:00 to 18:00 h) with controlled ambient temperature (23 °C ± 2 °C) and humidity (50–70%) and free access to water and food. Animals were allowed to adapt to the laboratory environment for 5 days prior to the start of the experiment.

**Figure 5:**
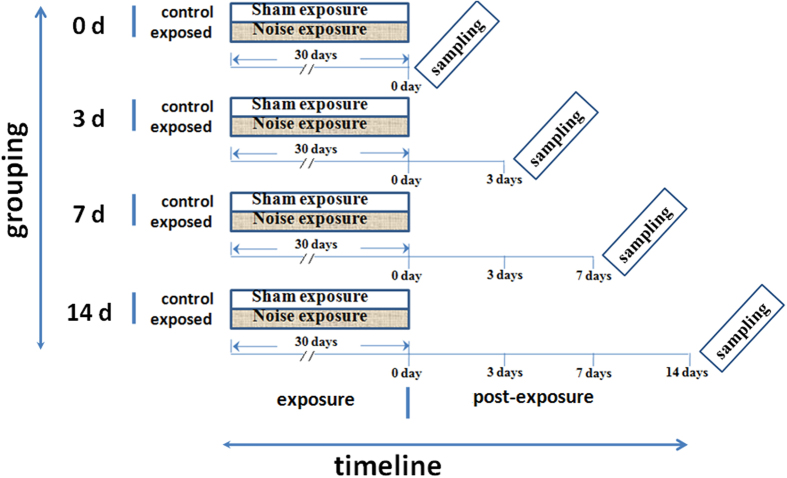
Experimental timeline. Animals were randomly assigned to 0, 3, 7, and 14 day groups according to when the end-point evaluation was performed (1, 3, 7, and 14 days following the final noise exposure, respectively), and each group were further subdivided into control and exposed subgroups, in which animals were subjected to 30 successive days of noise exposure (indicated by the gray area within the 30-day period). Animals in the control group were subjected to sham exposure (indicated by the blank segments in the 30-day period) for 30 days.

The rats were randomly assigned to 0, 3, 7, and 14 day groups according to when the end-point evaluation was performed (1, 3, 7, and 14 days following the final noise exposure, respectively), and each group was further subdivided into noise-exposure and control groups; the former was exposed to 100 dB sound pressure level (SPL) white noise (4 h/day for 30 days from 8:00 to 12:00 h), whereas control rats were housed in similar cages but exposed to background noise (<40 dB SPL) from another chamber. At different time points (days 0, 3, 7, or 14) after the last noise exposure, rats were sacrificed by decapitation, and blood, liver, intestine, cecum, and caecal content samples were immediately collected for biochemical analyses and stored at −80 °C until use (Fig. 5). At least three animals were used per treatment group and per time point in each experiment. All animal experiments were performed in accordance with approved guidelines and the protocol was approved by the Institutional Animal Use and Care Committee, Tianjin Institute of Health and Environmental Medicine.

### Noise exposure set-up

White noise was generated using a noise generator (BK 3560C; Brüel & Kjær Instruments, Nærum, Denmark), amplified with a power amplifier (Yong-Sheng Audio P-150D; The Third Institute of China Electronic Technology Group, Beijing, China), and delivered to a loudspeaker (ZM-16S; Tianjin Zenmay Electroacoustic Equipment Co., Tianjin, China). Animals were exposed to noise in a reverberation chamber in wire-mesh cages placed at the centre of the sound field. A loudspeaker was suspended directly above the cages. Noise levels were measured with a 3560 C-size front end and 4938 1/4′′ pressure-field microphones (Brüel & Kjær Instruments) placed at the same level as the animals’ ears using linear weights. Variation in noise level was <2 dB within the space available to the animal. The main spectrum of the noise emitted from the speaker was in the range of 400–6300 Hz (measured using 1/3 octave bands).

### Determination of blood glucose level

Blood was collected from the tail vein of each animal after an overnight (12 h) fast at each time point to determine blood glucose level using a Gold-Accu blood glucose test meter (Sannuo, Changsha, China).

### Determination of protein concentration by ELISA

Blood plasma CORT level, liver glycogen and TG levels, and TNF-α, IL-1β, and IgA levels in intestine homogenates were measured with ELISA kits (BlueGene Biotech, Shanghai, China) according to manufacturer’s instructions. Frozen rat liver and intestine samples were homogenised in ice-cold 1× phosphate-buffered saline (0.02 mol/l, pH 7.0–7.2) and total protein concentration was determined with the bicinchoninic acid assay (Boster, Wuhan, China). Glycogen and TG concentrations in the liver and inflammatory factor levels in the intestine were normalised to total liver or intestine protein concentrations, respectively, and the mean value of duplicate samples was taken as the final concentration for each animal.

### Western blot analysis

Western blotting was carried out as previously described^5^ using rabbit antibodies against the following proteins: IR (1:800), IRS-1 (1:800), p-AKT (1:800), GSK-3β (S9) (all 1:800), or glyceraldehyde phosphate dehydrogenase (GAPDH) (1:10,000) (all from Bioworld, San Diego, CA, USA); GAPDH was used as an internal reference standard. Secondary antibodies were peroxidase-conjugated goat anti-rabbit IgG (H + L) (ZSGB-BIO, Beijing, China).

### Sequencing of the 16S rRNA gene in microbiota

Total genomic DNA was extracted from samples using the cetyltrimethylammonium bromide/sodium dodecyl sulphate method. DNA concentration and purity were verified by 1% agarose gel electrophoresis. DNA was diluted to 1 ng/μl with sterile water and ~10 ng were used as a template to amplify the 16S rRNA gene in 30-μl reactions with 15 μl Phusion High-Fidelity PCR Master Mix (New England Biolabs, Ipswich, MA, USA) and 0.2 μM forward and reverse primers. Thermal cycling consisted of 98 °C for 1 min; 30 cycles of 98 °C for 10 s, 50 °C for 30 s, and 72 °C for 30 s; and 72 °C for 5 min. Equal volumes of 1× loading buffer (containing SYBR Green) and PCR product were mixed and resolved by 2% agarose gel electrophoresis. Samples between 400–450 bp were selected for further experiments. PCR products were mixed in equidensity ratios and purified using a GeneJET Gel Extraction kit (Thermo Fisher Scientific, Waltham, MA, USA). Sequencing libraries were generated using the TruSeq DNA PCR-Free Library Preparation kit (Illumina, San Diego, CA, USA) according to the manufacturer’s protocol, and index codes were added. The library quality was assessed with a Qubit@ 2.0 fluorometer (Thermo Scientific) and the Agilent Bioanalyser 2100 system. The library was sequenced on an Illumina HiSeq platform and 250-bp paired-end reads were generated. Sequences were analysed using Quantitative Insights Into Microbial Ecology (QIIME) software (http://qiime.org/). Reads were first filtered by QIIME quality filters. Pick_de_novo_otus.py was used to select operational taxonomic units (OTUs) with a table. Sequences with ≥97% similarity were assigned to the same OTU. A representative sequence was selected for each OTU and the Ribosomal Database Project classifier was used to annotate taxonomic information for each representative sequence^47^.

### Statistical analysis

Data presented in graphs indicate group mean ± standard deviation and were analysed using SPSS v.19.0 software (SPSS Inc., Chicago, IL, USA). Normality of raw data and residuals was tested with the Shapiro-Wilk test. Because of skewed distributions, the values of CORT content, blood insulin, and the liver TG level were logarithmically transformed (log_10_) for statistical analysis. Data obtained for the 16S rRNA gene in microbiota was assessed using the Wilcoxon rank-sum test. The Student t test was used to evaluate mean differences in all other data. P < 0.05 was considered statistically significant.

## Additional Information

**How to cite this article**: Cui, B. *et al*. Effects of chronic noise on glucose metabolism and gut microbiota-host inflammatory homeostasis in rats. *Sci. Rep.* **6**, 36693; doi: 10.1038/srep36693 (2016).

**Publisher’s note:** Springer Nature remains neutral with regard to jurisdictional claims in published maps and institutional affiliations.
